# *“I Would Have to Hold Him and Pin Him Down”*: Caregiver Experiences of Administering Drug-Resistant Tuberculosis Treatment to Children in KwaZulu-Natal, South Africa

**DOI:** 10.5334/aogh.5233

**Published:** 2026-07-15

**Authors:** Boitumelo Seepamore, Nirupa Misra, Brittney J van de Water, Jennifer Furin, Shriya Misra

**Affiliations:** 1 Discipline of Social Work, University of KwaZulu-Natal, Durban, South Africa; 2 King Dinuzulu Hospital Complex, Centralized Drug-Resistant Tuberculosis Hospital, Durban, South Africa; 3 Discipline of Health Sciences, University of KwaZulu-Natal, South Africa; 4 Connell School of Nursing, Boston College, Chestnut Hill, MA, USA; 5 Department of Global Health and Social Medicine, Harvard Medical School, Boston, Massachusetts, USA; 6 The Health Ninja Pty Ltd, Durban, South Africa; 7 Divine Health Forum NPC, Durban, South Africa; 8 SYNAPSE Research Collective, Wits Health Consortium, Pietermaritzburg, South Africa

**Keywords:** child-friendly formulations, acceptability, pediatric DR-TB, caregiver experiences

## Abstract

*Background:* The lack of child-friendly, second-line drug-resistant tuberculosis (DR-TB) medication in South Africa often leads to the use of adult formulations, which are not always suitable for children. Caregiver experiences in preparing and administering this treatment and the impact on parent–child relationships are poorly represented in the literature.

*Objective(s):* To assess parental acceptability regarding the preparation and administration of DR-TB medications for young children, utilizing Wademan’s acceptability framework.

*Methods:* Seven caregivers of eight children who had been diagnosed with DR-TB between June 2019 and February 2022 and initiated treatment at a referral hospital in KwaZulu-Natal participated in the study. Individual in-depth interviews with three caregivers and one focus group discussion with four caregivers were used to gather data, which were analyzed using part of the acceptability framework for TB in children developed by Wademan et al. [1].

*Findings:* Despite challenges, caregivers were adept at navigating DR-TB medication regimens, highlighting their critical role in inventive preparation methods to enhance palatability and strategic administration practices for children. Adult medications were adapted where necessary, and caregivers employed various techniques to ensure adherence. The acceptability of treatment was affected by medication palatability, preparation and administration, appeal and side effects (usability), and the interface between caregivers and the healthcare system (integration). Staying engaged in care threatened not only family resources but also the parent–child relationship. The daily struggle was between children often resisting medication and caregivers needing to find ways for the children to take medication, for instance, by begging, reasoning with, threatening, and forcing them.

*Conclusions:* Despite all formulations having adverse effects, caregivers and children preferred child-friendly formulations due to their ease of preparation and administration. Child-friendly formulations of DR-TB medications should be widely available and be the standard of care in all settings.

## Introduction

An estimated 1.17 million children developed tuberculosis (TB) in 2021, and an estimated 25,000–32,000 of these had drug-resistant tuberculosis (DR-TB) [2]. The diagnosis and treatment of pediatric TB are often complicated even more by the lack of child-friendly formulations and long treatment regimens requiring manipulation of adult TB medication [3, 4]. There are multiple barriers to successful treatment, including a lack of easy-to-administer diagnostic tools for children, high pill burden, medication side effects, and drug–drug interactions. Growing research indicates the need for more palatable, acceptable, and tolerable medications, particularly for children with DR-TB [4].

Palatability, defined as the organoleptic properties that include smell, taste, aftertaste, dose volume, and size, texture, or mouthfeel, may affect adherence [5, 6]. Coupled with this is acceptability—the ability of the patient and the caregiver to use a medicinal product as intended. Factors such as the shape, color, surface coating, and taste, smell, and texture of liquid, capsules, or tablets determine medication acceptability [7, 8]. Some forms of oral medication, such as dispersible and effervescent tablets and multi-particulates, may be better tolerated by children [8]. There is a growing need to undertake studies with target populations using standardized and harmonized tools as most pharmacological studies are undertaken in the adult population [9–11]. Targeted populations, such as children, become part of a therapeutic triad involving the child (patient), parent (caregiver), and healthcare professional, complicating the treatment regimen and may impact “normal” family life [12, 13].

Specifically, the experiences of caregivers who administer DR-TB medication to children are not well represented in the literature. The lack of clear dosing guidelines for infants in South Africa further complicates the treatment of children with DR-TB [13]. Doron, Hen, and Sharabi-Nov found that relationships with children were affected by “differences in disease-related demands, severity of the illness, prognosis, intensity of treatment, and predictability of exacerbations and side effects” [12]. The responses of parents could be overprotection or an expectation for increased independence in their children. Understanding the caregiver perspective enables family-centered care to be provided—such as including patients and caregivers in decision-making—and may improve the family and healthcare team’s experience and treatment outcomes [13].

While children are initiated on treatment and receive care from healthcare workers at the start of their treatment journey, the burden of care depends largely on caregivers, who are actively involved in the preparation and administration of medication for the remaining duration and completion of the treatment regimen at home [14–16]. Caring for children with chronic diseases may be challenging, yet some studies have found positive outcomes, such as caregivers feeling empowered when they confidently prepare and administer medication at home, establish a household routine, and gain a sense of accomplishment at treatment completion [17]. Complex regimens, therefore, require appropriate and acceptable interventions. Supporting caregivers of children with chronic diseases such as DR-TB is critical.

## Methods

### Participant selection and recruitment

This study assessed the acceptability of DR-TB treatment in purposively sampled caregivers of children who received a combination of adult and child-friendly formulations of DR-TB medication between June 2019 and February 2022. A total of 25 children under 6 years of age (who were eligible for the use of child-friendly formulation and were administered at least one child-friendly formulation DR-TB treatment) were identified from the patient TB register as having initiated treatment at a centralized DR-TB hospital in Durban, KwaZulu-Natal province, South Africa. A total of 24 caregivers were identified from the database, with 1 caregiver being responsible for 2 children who were on treatment. These 24 caregivers were potential participants to be recruited for this study.

**Figure 1:**
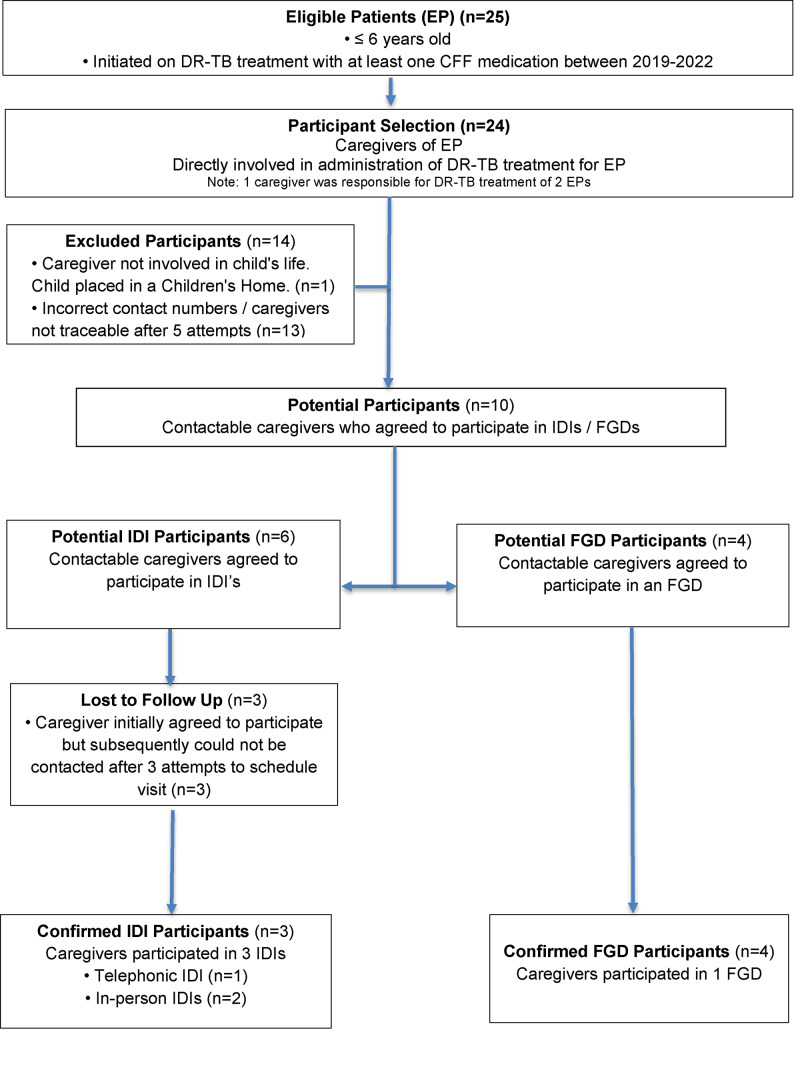
Figure of participant selection.

Potential participants were first contacted telephonically by study teams for a maximum of five attempts. If primary contact numbers were incorrect, patients’ hospital files were checked for alternative contact details, and the study team attempted to contact these alternate contacts for a maximum of five attempts. Failure to reach a potential participant after the fifth attempt resulted in exclusion. Figure 1 shows the recruitment process for potential participants, resulting in the final participants for this study, referred to as “caregivers” here-on-out. The option to participate in in-depth interviews (IDIs) was offered to caregivers who could not travel to the venue and/or were not available at the time of the focus group discussion (FGD). The length of the FGD was 1 hour, and the length of IDIs was approximately 30 minutes.

Caregivers (*n* = 7) of the eight patients/children provided informed consent and participated in once-off IDIs or FGD between August and October 2022. All caregivers who attended in-person interviews signed informed consent forms, and those who participated in telephonic interviews provided verbal consent. The informed consent process and interviews were offered in either English or isiZulu; all caregivers opted for these to be held in isiZulu and agreed to the recording of these IDIs/FGD. Caregivers were made aware that the study aimed to explore their experiences with both adult and child-friendly DR-TB medication that their children were prescribed.

Research assistants were trained by a qualitative research expert in conducting qualitative research and the use of interview guides to conduct both IDIs and FGDs in English and isiZulu. Recordings were sent to an independent, trained transcriber, who was not involved in the interviews, and who transcribed the audio recordings verbatim and translated them into English. The translated documents were reviewed by all authors and manually analyzed to extract themes.

## Data Collection

### Wademan framework of acceptability

The Wademan et al. acceptability framework and its three domains (usability, receptivity, and integration) for TB in children were used to analyze data [1]. Within this study, the ability of the child and caregiver to incorporate treatment into their daily lives by exploring palatability, administration, and medication appeal refers to the usability domain. Palatability includes the volume of the medication and whether the medication is mixed with other agents such as food [3]. Although pediatric formulations have traditionally been palatable, the taste, aftertaste, mouthfeel, and smell could affect acceptability. Coupled with this was the appeal of the medication, which refers to its color, size, packaging, storage, and simplicity of instructions. Further, administration, or the ease with which the medication can be prepared, was a major issue determining its acceptability and the relationship between caregiver and child. We also focused on the integration domain, particularly the interaction of caregivers with the healthcare system.

### Analysis

**Figure 2:**
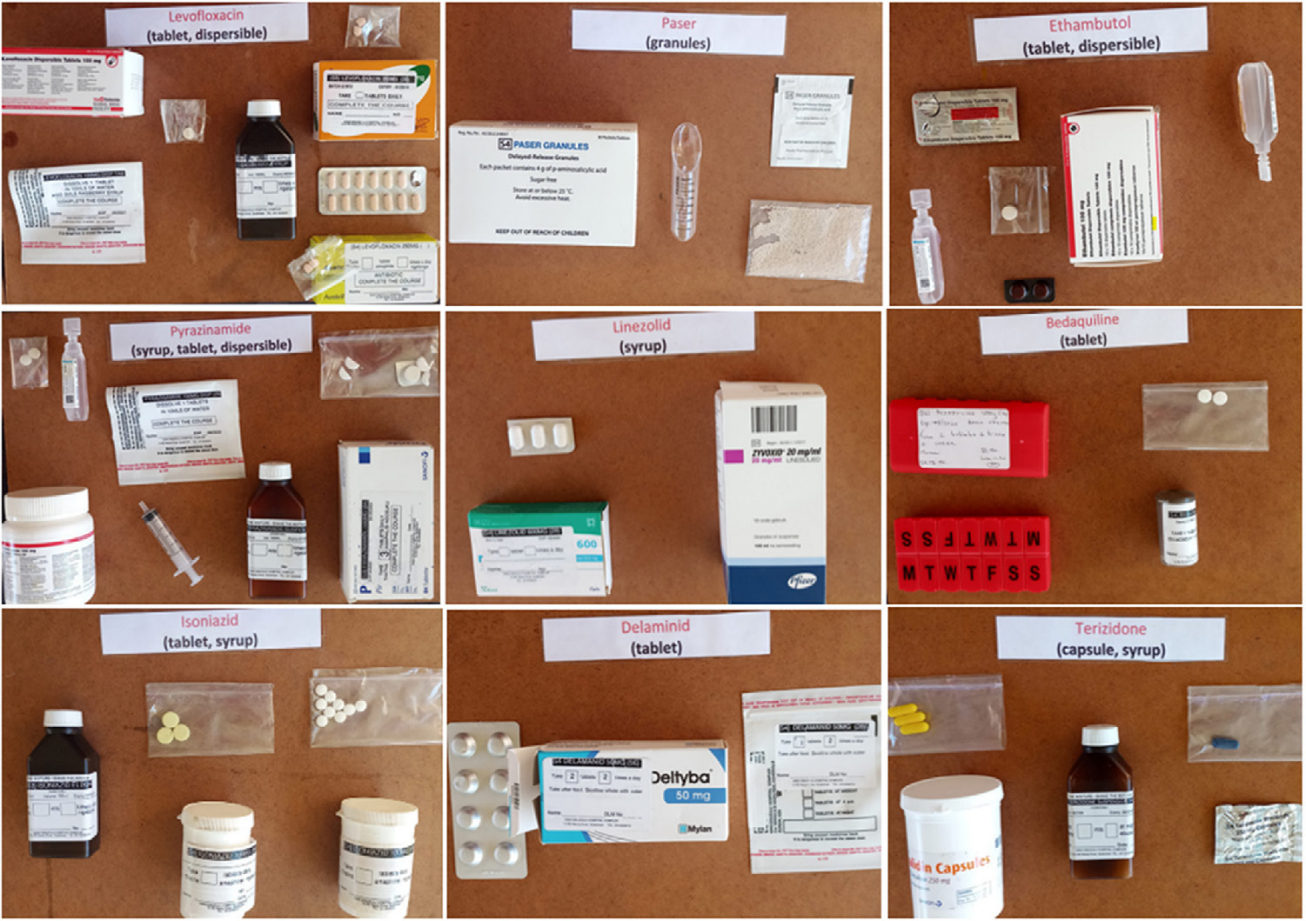
Photos of medications used as reference tools.

Medication reference tools (Figure 2) were developed, showing sample boxes of the medication, tablets, syrups, and administration tools, such as syringes, to remind caregivers of the medication that their child received during treatment. Different brands of DR-TB medication (that were available to patients during their treatment) were shown, including the novel child-friendly formulations, with external packaging and samples of the actual tablets and syrups, and labels with doses and names. Samples of the actual tablets, syrups, and containers were displayed with any new or different packaging, as shown in Figure 2.

In-depth interviews using semi-structured interview guides enabled the research team to engage caregivers fully, probe, and follow up on what was unclear. These guides were developed to suit both FGD and IDIs and were adapted accordingly for caregivers who expressed interest in individual discussions, due to time constraints or personal preference. Interviews were recorded, transcribed, translated, and analyzed using thematic analysis. This involves familiarizing the researcher with the data, coding, generating general themes, reviewing, and classifying them into categories, which are then defined and presented [18, 19]. Transcripts were analyzed by categorizing data into themes, guided by Wademan’s framework of acceptability, focusing on three domains and their respective dimensions that influence acceptability: (1) usability—or how easy treatment is to use/administer; (2) receptivity—or the expectations of the caregivers and children about treatment; and (3) integration—how well the treatment fits in with other aspects of the caregivers’ and children’s lives. The conceptual framework of the acceptability of tuberculosis treatment in children was used to generally guide the analysis of this small homogeneous target group, while also allowing other themes to be developed, or excluding a domain or dimension if unwarranted.

Caregivers who agreed to participate in the study gave written informed consent before the interviews and recordings. This study was approved by the Provincial Department of Health in KwaZulu-Natal as part of a larger mixed-methods study (KZ202008_059).

## Results

The seven caregivers were all biological mothers of the children whose ages ranged from seven months to five years. All caregivers lived with their children and were the main caregivers after discharge. The length of hospital admission for each child ranged from three weeks to six months. Of the eight children in the study, two were prescribed the long DR-TB treatment regimen while the rest were on the short DR-TB treatment regimen. Their regimens consisted mainly of adult formulations and at a later stage, after a donation of child-friendly formulation was received, a combination of manipulated adult formulations and child-friendly formulations. Some medications were in tablet form, while others had a dispersible or syrup alternative. Raspberry syrup or a commercially available multivitamin syrup, together with syringes, was also dispensed to each caregiver by the hospital. Table 1 details a qualitative data matrix highlighting illustrative quotes by theme.

**Table 1:** Qualitative data matrix.

ILLUSTRATIVE QUOTE	IDI V FGD
**Theme per Wademan’s Framework of Acceptability: Manipulation and administration of DR-TB medication**	
*“I was breaking it up and I was grinding it and put it in water and mix it with the… small white one and one red one and they would dissolve so I mixed them in a container…”*	FGD, P3, child 10 months
*“… ah, we give him in the morning, I used to give him at 4:H00 because…”*	IDI 3, child 7 months
*“I would wake up in the morning and prepare for my other children who were already in school, and so by six am I was already up and at that time I would give Paser also whilst busy tiding up the house the porridge would be cooking and at around 8 am I knew that I had to administer the rest of the medication before he ate porridge.”*	FGD, P3, child 10 months
*“my five-year-old can talk for herself, and she always says ‘will you buy Danone (yoghurt) or juice for me when I finish drinking my medication?’”*	IDI 1, child 5 years
*“When he said that it is bitter, I would put my hand in my pocket and promise to give him R5.00 ($0.27) and encourage him to take his medicines and… on Saturday, I would decrease the amount to R2.00 ($0.11) and we would build it up as the week goes.”*	FGD, P4, child 3 years
*“That’s the tablet that would change her demeanor, and sometimes she would even cry, and my uncle’s wife would help me to hold her.”*	IDI 1, child 5 years
*“He would change his face because they are bitter, so suddenly… he just doesn’t like them, he cries when he is going to be given these pills…”*	FGD, P3, child 10 months
*“I would have to hold him and pin him down.”*	IDI 3, child 7 months
*“I would use a syringe and just push it forcefully and tell him to swallow, and he would swallow very quickly, and maybe if I have some sweets next to me, then I would give it to him, but other times, he just drank his medication without a hassle.”*	FGD, P2, child 18 months
*“He used to say that it made his tummy hurt, and he felt tired and nauseous. He would beg me not to give [medication] to him or try to bargain with me that I give him his medication on the following day or later, he kept running from taking them.”*	IDI 2, child 3 years
*“…after taking it, he would refuse to eat, saying he may throw up, and he needs time and maybe he would eat around 10 am since the morning”*	IDI 2, child 3 years
*“I would wake up in the morning and prepare for my other children who were already in school*	
*“No, shame they are very bitter and so it was just a fight, the moment he woke up he was ready (for a fight), and I too would make haste to administer medication to him.”*	IDI 2, child 3 years
**Theme per Wademan’s Framework of Acceptability: Interface with the healthcare system: caregivers caught in the middle**	
*“… and it was painful to see him cry too when we had to leave him, it was just the worse and the most torturous experience ever.”*	FGD, P3, child 10 months
*“Hmm, I used to cry, and I cried a lot [it was it difficult] it was not easy at all.”*	IDI 1, child 5 years
*“Hospital staff asked us to come and witness how they administer medication so that we too as parents could see the struggle and learn how we will cope”*	FGD, P4, child 3 years
*“I was coming to this hospital and when I finally arrived, they treated us with dignity and gave me a bed to sleep in even though I came a day before I was expected to come. The next day the necessary tests were done, and I left…”*	FGD, P1, child 10 months
*“…I couldn’t see her; I called her until I found out that she had gotten used to the hospital; it was good for her, and she would tell me that she had aunties in the hospital who bathed her and taught her new things in life. I realized that I needed to let my heart be at peace and know that my child would be okay.”*	FGD, P4, child 3 years

### Manipulation and administration of DR-TB medication

Integrating or incorporating treatment into daily lives was a challenge for both caregivers and the children, as it meant a change in their routine. Caregivers first had to be able to identify medication, understand correct dosages, use different administering tools, and be able to store the medication safely. Previously, caregivers had administered adult formulations to their children before child-friendly alternatives were made available, and now would have to adapt these techniques to these newly available formulations. Since DR-TB medications were available in different forms, such as tablets, capsules, dispersible tablets, granules, or syrups, caregivers needed to adapt their techniques to administer these appropriately. Medication that was only available as adult formulation still had to be manipulated by breaking, splitting, crushing, and mixing with a medium such as water and/or syrup to assist in improving taste and texture.

*“I was breaking it up and I was grinding it and put it in water and mix it with the… small white one and one red one and they would dissolve so I mixed them in a container…”* (FGD, P3, child 10 months)

Administration of the medication involved the ease with which the medication can be prepared and given to the children. While children were hospitalized for treatment, nurses in the pediatric ward taught caregivers how to prepare and administer the medication. Once children were discharged, caregivers planned to administer some medication in the morning and some at night as directed. While most caregivers administered these medications very early in the morning, some administered them at different times during the day, depending on their schedule.

*“… ah, we give him in the morning, I used to give him at 4:H00 because…”* (IDI 3, child 7 months)

“*I would wake up in the morning and prepare for my other children who were already in school, and so by six am I was already up and at that time I would give Paser also whilst busy tiding up the house the porridge would be cooking and at around 8 am I knew that I had to administer the rest of the medication before he ate porridge.”* (FGD, P3, child 10 months)

Different strategies were developed to aid medication administration (for caregivers) and/or to facilitate ingestion (for children). Caregivers would coax, beg, bribe, threaten, or physically restrain children to ensure treatment adherence. While some caregivers reasoned with children, others distracted them by watching TV or telling stories. Bribery included promising the child money, treats (sweets, chocolates, chips), toys (bicycles), or an outing to a favorite place or restaurant.

*“my five-year-old can talk for herself, and she always says ‘will you buy Danone (yoghurt) or juice for me when I finish drinking my medication?’”* (IDI 1, child 5 years)

*“When he said that it is bitter, I would put my hand in my pocket and promise to give him R5.00 ($0.27) and encourage him to take his medicines and… on Saturday, I would decrease the amount to R2.00 ($0.11) and we would build it up as the week goes.”* (FGD, P4, child 3 years)

Although most children could not verbally communicate a dislike for the medication due to their young age, caregivers reported that their children showed facial expressions of dislike, cried, or outright refused to take the medication. Child resistance to treatment was a constant battle which affected the caregiver–child relationship and led to caregiver fatigue. Just seeing the medication being prepared for administration resulted in some children reacting negatively.

*“That’s the tablet that would change her demeanor, and sometimes she would even cry, and my uncle’s wife would help me to hold her.”* (IDI 1, child 5 years)

*“He would change his face because they are bitter, so suddenly… he just doesn’t like them, he cries when he is going to be given these pills…”* (FGD, P3, child 10 months)

Caregivers also described difficult situations and inventive ways of making the children take the medication; one caregiver said: *“I would have to hold him and pin him down.”* (IDI 3, child 7 months)

Yet others reported cooperation, saying, *“I would use a syringe and just push it forcefully and tell him to swallow, and he would swallow very quickly, and maybe if I have some sweets next to me, then I would give it to him, but other times, he just drank his medication without a hassle.”* (FGD, P2, child 18 months)

To avoid taking the medication, some children would feign sleep, negotiate not to take the medication, or make the parents promise a treat.

*“He used to say that it made his tummy hurt, and he felt tired and nauseous. He would beg me not to give [medication] to him or try to bargain with me that I give him his medication on the following day or later, he kept running from taking them.”* (IDI 2, child 3 years)

Side effects played a major role in acceptability. Children experienced side effects such as nausea, fatigue, vomiting, and diarrhea. Caregivers also noted a change in skin color (from Clofazimine), and associated Bedaquiline with fatigue, while Para-aminosalicylic acid (PAS) was associated with diarrhea. The children were averse to other medication due to their (bitter) taste, such as Terizidone and Levofloxacin. However, not all medication tasted bitter. One caregiver described Isoniazid as tasty with a *“minty-ish”* caramel taste. Caregivers reported various side effects from the medications, for instance:

*“…after taking it, he would refuse to eat, saying he may throw up, and he needs time and maybe he would eat around 10 am since the morning”* (IDI 2, child 3 years)

Some medicines such as PAS, which came in multi-particulate form, had to be given first thing in the morning, and administered with a sour medium such as yoghurt, juice, or *amasi* (sour milk). This meant that some children had to be woken up hours before their usual time. Some caregivers administered it at least 30 minutes to 1 hour before other medications to prevent nausea and vomiting and ensure medication absorption. This disrupted the usual family routine, especially in the morning when the caregivers were getting themselves ready for the day and busy with other caregiving duties.

*“I would wake up in the morning and prepare for my other children who were already in school and so by six am I was already up and at that time I would give PAS also whilst busy tiding up the house the porridge would be cooking and at around 8 am I knew that I had to administer the rest of the medication before he ate porridge.”* (FGD, P3, child 10 months)

Caregivers prepared medications in the presence of children to help signal “medication time,” which led to an unpleasant atmosphere in the household.

*“No, shame they are very bitter and so it was just a fight, the moment he woke up he was ready (for a fight), and I too would make haste to administer medication to him.”* (IDI 2, child 3 years)

### Interface with the healthcare system: Caregivers caught in the middle

Caregivers were in a complex relationship between building relationships with healthcare workers and threatening their relationship with their children. This paradox was presented by the necessity to leave their children with strangers and wanting their children to get better despite the children’s protest. Although the children were patients, by virtue of being their caregivers, caregivers interacted with the healthcare system on behalf of the children as their guardian. Both caregivers and children responded differently to separation from one another, especially in instances of long hospital stays. Both showed distress around being separated.

*“… and it was painful to see him cry too when we had to leave him, it was just the worse and the most torturous experience ever.”* (FGD, P3, child 10 months)

*“Hmm, I used to cry, and I cried a lot [it was it difficult] it was not easy at all.”* (IDI 1, child 5 years)

All participants reported receiving good care in public facilities. They were able to build a good relationship with the pediatric nurses who took care of their children while admitted. Before the children were discharged, nurses showed caregivers how to prepare and administer the medication to children.

*“Hospital staff asked us to come and witness how they administer medication so that we too as parents could see the struggle and learn how we will cope”* (FGD, P4, child 3 years)

Although leaving children in the hospital was an unpleasant experience, caregivers described being satisfied with the care they and the children received. Some caregivers could sleep over for a day or two at the hospital where their children were admitted. Despite understanding that hospital staff were taking good care of their children and teaching parents how to properly administer medications, caregivers described the trust they had to have in the healthcare system and individual providers caring for their children.

*“I was coming to this hospital and when I finally arrived, they treated us with dignity and gave me a bed to sleep in even though I came a day before I was expected to come. The next day the necessary tests were done, and I left…”* (FGD, P1, child 10 months)

*“…I couldn’t see her; I called her until I found out that she had gotten used to the hospital; it was good for her, and she would tell me that she had aunties in the hospital who bathed her and taught her new things in life. I realized that I needed to let my heart be at peace and know that my child would be okay.”* (FGD, P4, child 3 years)

## Discussion

The difficulty of a long and complex treatment regimen did not always fit into the family’s normal routine, which, in some instances, threatened adherence and the relationship between children and their caregivers [12, 20]. Despite these challenges, caregivers wanted their children to be cured and showed commitment to the treatment course. However, the lack of child-friendly formulations made adherence to treatment more difficult [4, 6]. Preparation and administration of the medication were challenging despite being shown how to prepare and administer the medication by healthcare workers before the children were discharged, but this was not always possible to replicate at home.

Administering medication early in the morning meant that some children had to be woken up as early as two hours before the usual wake-up time, which not only disturbed the child’s sleep but also changed the caregiver’s schedule. Most caregivers administered complex medication routines while still managing their usual caregiving duties. The tuberculogenic environment refers to the complex interplay of broad environmental factors (physical, socioeconomic, commercial, political, and healthcare) and vulnerabilities, such as inadequate housing, food insecurity, and comorbidities, that increase an individual’s risk for TB and create barriers for successful treatment [21]. One solution is to integrate family-centered care interventions across the TB care cascade to engage and support families systematically throughout their treatment journey [13].

Medication preparation and administration were time-consuming, fraught with constant resistance from children and pressure to ensure adherence and correct dosing. The taste and side effects of the medications also affected acceptability. Because the children in this study were very young and may not have been able to understand or cooperate in their treatment or verbalize their distaste, their reactions to the medications were based mainly on facial expressions [8, 22, 23], which caregivers indicated as aversion to the medication.

Although sugar has traditionally been used to make pediatric formulations more palatable, this is no longer the practice as suggested by regulatory and professional agencies in the USA and Europe [7]. In one study, the bitterness of four anti-TB medications, namely rifampicin, ethambutol, pyrazinamide, and isoniazid, was tested, and the worst-tasting were ethambutol and isoniazid [8]. Similarly, children in this study did not like the taste of the medication, and caregivers described children showing resistance through crying, making faces, changing in demeanor, feigning sleep, or outright refusal. This affected the acceptability of medication and made administration difficult for caregivers.

Side effects and levels of toxicity may hinder adherence and can cause mild to serious side effects, such as vomiting, nausea, or gastrointestinal upset, peripheral neuropathy, optic neuritis, and psychological disorders [7, 24]. Some new and repurposed medications can be better tolerated in children with short-term use, but not longer regimens with numerous side effects.

Caregivers described interactions with the public healthcare system as generally positive, despite a preference for private healthcare [25]. TB diagnosis at public hospitals is routine and treatment for DR-TB is provided free of charge. The caregivers had trust in the treatment regimen and sought care from public health facilities as soon as children showed symptoms of illness. The separation of children from their caregivers was difficult for both participants and their children. Similarly, Meyerson et al. found that hospital stays brought about a range of behaviors such as frequent crying, aggression, hyperactivity, and withdrawal on the part of the children. Caregivers had to resort to deception, threats, and a prioritization of biomedical health over psychological health [26]. At times, caregivers had to make the decision to leave their children in the hospital even when both mother and child found this distressing.

In this study, caregivers reported high support from healthcare workers. They were given health literacy support, were shown how to prepare and administer medication at home, and were allowed to sleep over at the hospital, especially when the children had just initiated treatment. Health literacy is critical as it has been shown to be associated with TB treatment adherence and positive treatment outcomes [27].

## Limitations

This study had a very small sample, which offered insights aligned with Wademan’s framework, specific to caregivers and children. It contributes to the understanding of how existing theoretical constructs apply in a specific context. Caregivers who participated in this study were mainly Black women from a low socio-economic background who were either unemployed or working in the informal sector, and their children were receiving treatment from a large government DR-TB hospital. This homogenous sample may have introduced selection bias and is not representative of the larger community. As a result, the findings presented here may be limited in their generalizability, despite the ability to obtain rich information from caregivers, which can be used for further studies or to inform policy and practice. A contributing factor to a low response rate is that FGDs/IDIs were hosted during usual business hours, making attendance difficult for working-class caregivers. Additionally, some caregivers were interviewed in a group; therefore, responses may have been influenced by “groupthink,” where a group member conforms to the majority opinion rather than stating their own [28]. Given this small sample to conduct interviews from, the ability to reach conceptual saturation was limited and ideally more FGDs and IDIs could have been conducted with those unable to attend or with a wider representation of caregivers [29]. All caregivers were interviewed in a language of their choice, and they preferred isiZulu, which was also spoken by the interviewers. Translating the interviews into English and transcribing the data may have diminished the impact of some of the statements that are best understood in isiZulu and thus may have “neutralized” some data [28].

## Conclusion

Understanding the challenges associated with preparing and administering medication to children younger than six years is essential, and rests heavily on the relationship between caregivers, their children, and their interaction with healthcare workers. While advances have been made with new and repurposed DR-TB medications, there is a need for ongoing research to develop easier and more palatable medications for children to drive down rates of DR-TB in children to ensure successful outcomes. Clear preparation and administration guidelines, including innovative tools for correct dosing, if manipulated, must be urgently rolled out and made available for easier administration at home. More research is needed to fully understand and make recommendations to overcome the challenges experienced by caregivers and children with DR-TB.
